# Eradication of HCV in Renal Transplant Recipients and Its Effects on Quality of Life

**DOI:** 10.1155/2018/8953581

**Published:** 2018-08-30

**Authors:** Massimo Sabbatini, Ivana Capuano, Silvia Camera, Lucia Ferreri, Pasquale Buonanno, Laura Donnarumma, Nicola Caporaso, Filomena Morisco

**Affiliations:** ^1^Dipartimento di Salute Pubblica, Università degli Studi Federico II di Napoli, Italy; ^2^Dipartimento di Medicina Clinica e Chirurgia, Università degli Studi Federico II di Napoli, Italy; ^3^Dipartimento di Scienze Riproduttive ed Odontostomatologiche, Università degli Studi Federico II di Napoli, Italy

## Abstract

**Background:**

The use of direct antiviral agents (DAA) has radically modified the course of HCV hepatitis in renal patients. Aim of this study was to assess the effects of HCV eradication on quality of life (QOL) in renal transplant recipients (RTR), measured by CLDQ and SF-36.

**Methods:**

Sixteen RTR with well preserved GFR (mean: 60.3±19.3 ml/min) and chronic HCV infection with moderate liver stiffness (9.3±1.7 kPa) were given a sofosbuvir-based regimen for 12 weeks and had a 1 year follow-up.

**Results:**

At end of treatment (EOT) a complete viral clearance was observed in all the patients, with normalization of most laboratory data and a consistent reduction in liver stiffness. All these parameters remained stable after 1 year, as well as renal function and proteinuria. Questionnaire data showed consistent amelioration in different “emotional” domains at EOT, which persisted after 1 year and were associated with a globally improved QOL, although there was no change in most of the “physical” domains in both questionnaires. One patient under ribavirin developed an acute anemia and withdrew from the study, but no further adverse episode was observed throughout the study.

**Conclusions:**

Our data, while confirming the efficacy of oral DAA, show that HCV infection represents a heavy psychological burden in renal transplant recipients, greatly alleviated by viral eradication, which determines a significant improvement in QOL that represents an important outcome in management of all transplant recipients. This trial is registered with ISRCTN97560076.

## 1. Background

Chronic hepatitis by C virus infection (HCV) is a significant public health problem with a worldwide estimated prevalence of 3% [1], consistently higher in patients with chronic kidney disease (CKD), either in conservative and substitutive treatment [2]. In renal transplant recipients (RTR), HCV infection is associated with a significantly higher risk for all-cause mortality and graft loss compared to the uninfected counterpart [1, 3] and represents a serious trouble for these patients, due to the negative impact of immunosuppression on disease progression and its effect on the outcome of their graft, as a consequence of increased incidence of diabetes, enhanced onset of cardiovascular diseases, easier recurrence of glomerulonephritis, and faster progression of chronic allograft nephropathy [4–8].

The recent introduction of second-generation direct antiviral agents (DAA) has dramatically changed the therapeutic scenario of HCV infection in general population, as well as in renal patients [9, 10], and mostly in RTR, in whom interferon-based regimens are contraindicated because of the risk of acute graft rejection [8]; indeed, these new regimens have evidenced an optimal response in terms of either viral clearance or patients' tolerability [2, 11–18].

Given the high impact of HCV infection on patients' quality of life (QOL) in general population [19], the aim of the present study was to evaluate the effects of HCV eradication on health-related QOL in RTR, commonly affected by a heavy clinical and psychological burden. Today, improvements in QOL represent a widely accepted measure of treatment outcome in any chronic disease, since patients require greater attention to their physical and emotional well-being in everyday life.

To this extent a small cohort of RTR underwent a 12-week sofosbuvir-based regimen and was prospectively followed up for one year, with repeated evaluation of QOL questionnaires. Our working hypothesis was that eradication of HCV may determine consistent improvements in QOL.

## 2. Materials and Methods

### 2.1. Patients

This case series consisted of 16 HCV-infected kidney transplant recipients in regular follow-up at our Nephrology and Kidney Transplant Unit, referred to our hepatologists (NC, FM), who selected the proper DAA combination on the basis of HCV genotype and estimated glomerular filtration rate (eGFR) and drug availability in Italy at time of starting treatment. Our transplant recipients were white-Caucasian, all being first transplant from cadaver donors (two with simultaneous renal and liver transplantation).

The first 10 patients enrolled in the study were selected among RTR in regular follow-up at the Day Hospital of Renal Transplantation of University Federico II of Naples, on the basis of specific criteria suggested by Italian Ministry of Health in September 2015. They were as follows: age ≥18 years; presence of HCV-antibodies and of HCV-RNA replication, independently of liver enzymes alteration; a liver stiffness value ≥7 kPa at transient elastography (TE); presence of HCV genotype 1, 2, 3, or 4; and stable renal function in the last 6 months, with an estimated glomerular filtration rate (eGFR) >35 ml/min and no graft rejection in the preceding 12 months. From September 2016 DAA treatment could be started also in patients with lower TE values, and 6 further patients were enrolled. Exclusion criteria were as follows: decompensated liver cirrhosis, chronic B-hepatitis or human immunodeficiency virus infection, and presence of specific intercurrent clinical problems, like infections or ESA-resistant anemia.

### 2.2. Treatment

Antiviral therapy consisted of sofosbuvir (400 mg/day) in all patients, associated with daclatasvir (60 mg/day; n=9), ledipasvir (90 mg/day; n = 2), ribavirin (weight-based dosage; n=2), or velpatasvir (100 mg, n=2); one patient was under sofosbuvir + ledipasvir + ribavirin. The addition of ribavirin and of its discontinuation was at the discretion of hepatologist. Treatment lasted 12 weeks, like in general population, and its efficacy was evaluated by monitoring viral load at baseline, after 4, 8, and 12 weeks of treatment (end of treatment, EOT), and 12 and 48 weeks after EOT. Liver stiffness was evaluated by TE [FibroScan; Touch 5.02; Echosens; France] at baseline, at EOT, and 48 weeks after EOT by a skilled operator (SC), on the basis of current guidelines. Maintenance immunosuppression therapy consisted of a calcineurin inhibitor (CNI) in 12 patients (5 on tacrolimus and 7 on cyclosporine) in conjunction with steroids (n=9), mycophenolic acid derivatives (n=6), and sirolimus (n=2). Four patients were treated with sirolimus in association with steroids (n=4) and mycophenolic acid derivatives (n=2). In all the patients, induction therapy consisted of Basiliximab (20 mg, during surgery and at the 4th postoperative day).

### 2.3. Quality of Life Measurement

All the patients were administered the Chronic Liver Disease questionnaire (CLDQ) and the Short Form Health Survey (SF-36) questionnaire during their clinical visits, i.e., before starting the therapy (Basal), at the end of therapy (EOT), and 48 weeks later (1 year). CLDQ is the first disease specific instrument developed to evaluate health-related quality of life (QOL) in patients with liver disease [20] which includes 29 items in the following domains: fatigue, activity, emotional function, abdominal symptoms, systemic symptoms, and worry. The answers to each question are graded with scores ranging from 0 (best option) to 6 (worst option), according to its Italian version [21]: high scores denote a worse liver-related quality of life.

Health-related QOL was evaluated using the Italian version of SF-36. This tool contains one item that evaluates the perceived changes in health status, while the remaining 35 items are used to generate eight subscales, which are the weighted sums of the questions in their section. Each scale is directly transformed into a 0-100 scale on the assumption that each question carries equal weight; the lower scores reflect greater disability. The eight sections are general health perception, physical functioning, role limitation due to poor physical health, role limitation due to poor emotional health, social role functioning, bodily pain, emotional well-being, and vitality. Both questionnaires were given and explained to patients in the early morning of the scheduled visit by their trained caregiver (IC) and were completed at her presence after the visit.

### 2.4. Laboratory Data

Trough levels of tacrolimus, cyclosporine, and sirolimus were monitored every week during DAA administration and every 4 weeks later on by commercial immunoassays. Estimated-GFR (eGFR) was calculated with the Chronic Kidney Disease Epidemiology Collaboration Equation (CKD-EPI). Urinary protein excretion was measured on 24-hour urine samples.

All laboratory values were determined by standard methods. Plasma HCV mRNA levels were measured by a real-time PCR-based method (Abbott Real Time, Lower Limit of Quantification: 12 IU/ml).

### 2.5. Statistical Analysis

Data were first analyzed with Shapiro's test to determine their distribution. Parametric data were presented as mean and standard deviation; nonparametric data were presented as median and range. Parametric data were analyzed with repeated measures ANOVA or Student's t test for paired data, as appropriate; nonparametric data analysis was performed by Friedman's test. Post hoc analysis was conducted by Bonferroni's test for parametric data and Dunn's test for nonparametric data. Differences were considered statistically significant if p<0.05. Data were analyzed using statistical software R (R version 3.3.3).

The study was approved by Ethical Committee of “Federico II” University (#290/15). At time of starting treatment, all the patients were adequately informed about the potential adverse effects of DAAs and their possible interactions with immunosuppressive drugs; all the patients gave their informed written consent. Data were collected from October 2015 to February 2018.

## 3. Results

Baseline patients' demographic and clinical data are summarized in Table 1. Six patients had a FibroScan value >10 kPa at time of starting therapy. All the patients had documented HCV infection prior to transplantation and received HCV-negative organs. Their median HCV-RNA concentration was 2.1E^6^ (4.1E^4^-1.3E^7^) log 10 IU/mL, and a viral load greater than 800,000 IU/mL was present in 14 patients. Mean plasma concentrations of liver enzymes were in the upper zone of normal ranges.

The etiology of renal disease was undetermined in 57% of patients; six patients had biopsy proven glomerular diseases (2 focal and segmental glomerular sclerosis, 1 IgA-nephropathy, 1 postinfectious, and 2 not defined), and proteinuria exceeded 1 g/24 hours in 2 patients; 3 patients were affected by posttransplant diabetes (one insulin-dependent). One patient withdrew from the study during the 4th week of treatment because of acute anemia and his data are not considered in statistics; the remaining 15 patients completed at least 1-year follow-up. At baseline, no correlation was detected between FibroScan values, viral load, and liver enzymes (P=0.14).

### 3.1. Effects of DAA on HCV Infection 

**Figure 1:**
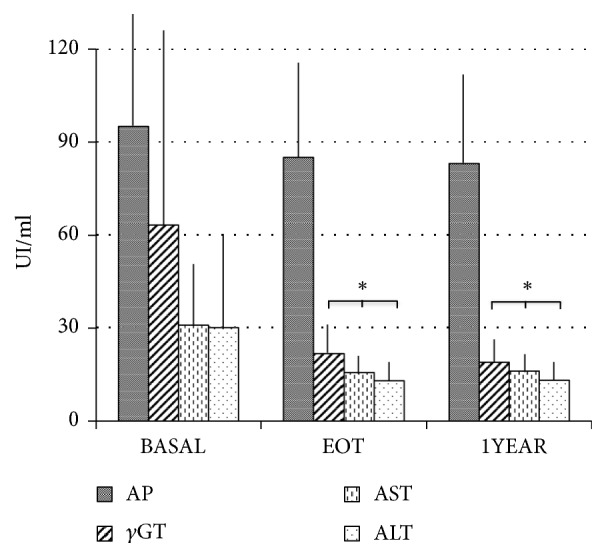
Main liver enzymes plasma concentrations in Basal condition, at end of therapy (EOT) and 1 year after EOT (n=15). Abbreviations. AP: alkaline phosphatase; *γ* GT: *γ*-glutamyl-transpeptidase; AST: aspartate aminotransferase; ALT: alanine aminotransferase. Data are expressed as mean values ± SD. *∗* means p<0.05 AST/ALT/GGT versus respective Basal value (p<0.05, minimum value).

**Figure 2:**
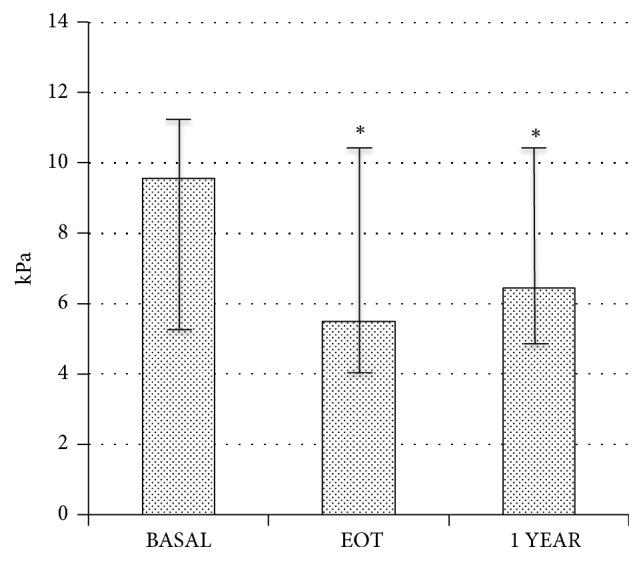
Modifications of transient elastography data (FibroScan) at end of antiviral therapy (EOT) and after 1 year compared to Basal (n=15). Data are expressed as median values and range (error bars). *∗* means p<0.0001 versus Basal.

A complete viral clearance, i.e., undetectable HCV-RNA replication, was observed in all the patients at EOT. It was associated with a significant decrease of GGT, AST, and ALT values (-60%, -49%, and -57%, respectively, versus Basal), with no change in alkaline phosphatase nor in total bilirubin (Figure 1); these parameters remained stable throughout the follow-up. Interestingly, also FibroScan values were consistently reduced at EOT (-33%) and were not modified thereafter (Figure 2), implying that no further improvement was obtained in the medium term despite a better functioning liver. No change was detected in hemoglobin, albumin, and glucose plasma levels throughout the study (data not shown).

### 3.2. Effects of DAA on Renal Function

DAA administration did not affect renal function. No change, in fact, was observed in mean eGFR at EOT (59.9±16.8 versus 60.3±19.3 ml/min in Basal, NS) nor after the 1-year follow-up (56.0±19.9 ml/min, NS), although two patients had developed an important impairment of eGFR (-31% and -49%, versus respective Basal). Similarly, proteinuria values remained quite stable throughout the observation period, averaging 0.58±0.76 g/24hrs at Basal, 0.55±0.77 at EOT, and 0.47±0.88 after 1 year (NS). Indeed, we observed an improvement in urinary protein excretion in 7/15 patients (with complete disappearance of proteinuria in 3 patients) and a clear worsening in the 2 patients who developed the renal impairment.

Trough levels of CNI were slightly reduced (tacrolimus, -24%; cyclosporine, -11%), and mild adjustments of drug doses were necessary in 3 patients. Sirolimus plasma concentrations, conversely, remained stable. Daily doses of mycophenolic acid and steroids were not changed during the study. The need for recombinant erythropoietin support or treatment of established diabetes also remained unchanged during therapy.

### 3.3. Quality of Life

**Figure 3:**
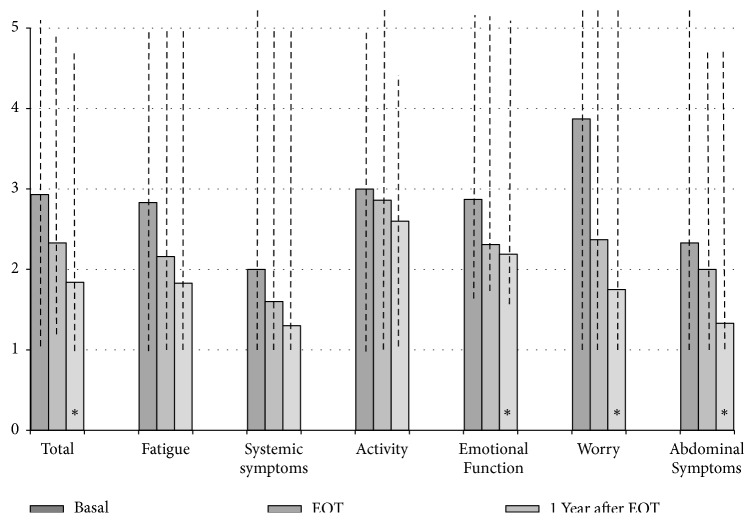
Scores of Chronic Liver Disease questionnaire (CLDQ) before starting treatment (Basal), at the end of therapy (EOT), and 1 year after EOT. Data are expressed as median values (range, dashed lines). *∗* means p<0.05 versus Basal (Friedman's test followed by Dunn's post hoc analysis).

HCV eradication was associated with a better QOL. At baseline, median value of CLDQ was 2.89 (range: 1.18-5.36), which denotes a relatively preserved liver-related quality of life. All the domains tended to be lower at EOT, although not significantly, with the exception of “activity”. At completion of the 1-year observation period (Figure 3), a consistent and significant decrease was recorded in global score, whose median value decreased by 36% (p<0.01 versus Basal), following the significant improvements observed in “worry”, “emotional function”, and perceived “abdominal symptoms”. Conversely, the domains of “fatigue”, “systemic symptoms”, and “activity” were not affected by HCV infection recovery.

The results of SF-36 questionnaire are reported in Table 2. It is interesting to note that both the scores of the “emotional well-being” and of the “role limitation due to emotional problems” were already significantly higher at EOT and further improved after 1 year, where also the perception of a beneficial “health change” was described, associated with a better perception of patient's social role. Interestingly, like in CLDQ, physical functioning and role limitation due to physical problems or vitality were marginally affected by DAA treatment.

### 3.4. DAA Side Effects

One patient developed a symptomatic episode of acute anemia during the 4th week of treatment with sofosbuvir + ribavirin (nadir of hemoglobin: 6.6 g/dl), which required hospitalization, transfusion of 2 blood units, and DAA withdrawal; the patient refused a new treatment with different drugs. Throughout the entire study, tolerance to treatment was excellent: mild headache (n=2) and fatigue (n=3) were the most common reported side effects, requiring no therapy. No acute graft rejection or infectious episode occurred in any patient, and after the initial decline, trough levels of immunosuppressive drugs remained stable during the follow-up period.

## 4. Discussion

In this paper we describe our single-center experience with a 12-week course of DAAs on 16 renal transplant recipients affected by long lasting HCV infection, with moderate liver stiffness and a well preserved renal function. Our data confirm the prolonged efficacy of DAAs in clearing viral infection and show that HCV eradication is associated with an early and persistent amelioration of self-perceived QOL.

### 4.1. Liver and Renal Data

All the modifications observed in liver function occurred during the 12 week treatment, and no further change was observed within the first year; similarly, hepatic stiffness greatly decreased at EOT (-33%) and remained quite stable thereafter: this huge reduction probably reflects the early improvement in liver necroinflammation induced by antiviral treatment [22].

HCV eradication did not modify mean eGFR nor proteinuria; it is noteworthy, however, that an important loss of filtrate was observed in 2 patients: the first one (combined liver/kidney transplantation) had a low baseline GFR and a high proteinuria (1.9 g/24hrs) at time of starting sofosbuvir + daclatasvir, which further increased to the nephrotic range despite the worsening of GFR (-49.5% at 1 year); the second one, conversely, presented a well preserved renal function and a proteinuria of 0.91 g/24hrs before starting sofosbuvir + ledipasvir. While, generally, our data provide confidence that DAA do not substantially affect renal function, it remains to be elucidated whether a preexisting, consistent proteinuria may predict the worsening of renal function after treatment. Our data, however, recall those of previous studies describing an unexplained early fall of eGFR in a small number of patients [11, 13, 23].

### 4.2. QOL Data

Undoubtedly, the most interesting aspect of the present study is the positive effect of HCV eradication on patients' QOL. Both questionnaires, in fact, evidenced interesting ameliorations in several domains, mostly relevant to the psychological sphere of our patients. At EOT, in fact, SF-36 data showed that both the well-being and the feeling of role limitation due to emotional problems were significantly better than those in Basal, and such improvements persisted after one year, associated with a healthier functioning role and a reduction in worry and emotional functioning observed with the CLDQ: taken together, all contributed to the positive perception of a beneficial modification in their QOL. Physical domains, conversely, were less affected by viral eradication; this result was in part expected, considering the long duration of HCV infection, which had probably reduced the intensity and the perception of symptoms, and the mild clinical expression of liver disease (no patient was cirrhotic).

The changes in QOL scores and, mostly, the rapidity of their improvement after DAA treatment clearly witness the heavy psychological burden that HCV infection determines in transplant recipients, who are obviously aware of the negative impact of immunosuppression on disease progression and fear the negative effects of a decompensated liver disease on the outcome of their graft; the entity of such improvement is not negligible considering that among solid organ transplant patients, kidney recipients show the lowest improvement in SF-36 after transplantation [24]. Although the questionnaires were not anonymous, but were proposed by their physician, we believe that patients did not overemphasize their feelings: all had a long follow-up in our unit and a complete confidence with their caregiver.

These data deserve attention since today QOL represents a crucial point in management of transplant recipients. In fact, traditional graft outcomes like organ survival, rejection rates, or transplant complications represent just a part of patients' concerns, and increasing attention is devoted to QOL: patients are more and more interested in how well they feel in “real life”, considering how much the disease has modified their lives. Therefore, although QOL measurements are based on patients' subjective sensations, they become a significant clinical measure, and patient's perspective becomes as important as that of the clinician [25]. Unfortunately, many physicians are still reluctant to consider improvements of QOL as a main outcome of transplantation.

In general population, two different reports describe improvements in QOL after DAA treatment [19, 26]. These data, quite surprisingly, were not confirmed by Ichikawa et al. in a cohort of cirrhotic patients that showed significant ameliorations in many cirrhosis-related symptoms after treatment [27]. Probably, the life expectancy of cirrhotic patients (compared to transplant recipients) and, mostly, the degree of hepatic impairment have conditioned this result: in fact, it may require several years after relief from liver-related symptoms for an effect on QOL to become apparent [27].

Tolerability of our sofosbuvir-based regimen was excellent, as also indirectly confirmed by the improved QOL. The only serious adverse effect was an acute episode of anemia probably related to the use of ribavirin; unfortunately, the patient refused a new therapeutic trial with different drugs. The good safety profile of DAA should alert physicians to treat potential candidates as soon as possible, while on dialysis or in the transplant waiting list, to prevent further liver deterioration and improve a depressed QOL.

The main limit of our prospective study is the small sample size, due to difficulties in obtaining the drugs from our Health National System, and the inclusion of only patients with moderate liver dysfunction. Our data, however, suggest that greater improvements of QOL could be expected in patients with more pronounced symptoms and greater psychological involvement.

## 5. Conclusions

In conclusion, our study demonstrates that, beyond the clinical results on liver disease progression, HCV eradication by DAA determines a consistent improvement in QOL of transplant recipients. Although new studies are necessary to evaluate whether the early HCV eradication will result in improved graft outcomes, decreased recurrence of glomerulopathies, or reduced incidence of posttransplant diabetes, the possibility of improving patients' QOL represents a further incentive to early treat all HCV-infected patients.

## Figures and Tables

**Table 1:** Patient characteristics at baseline (n=16).

**Demographic characteristics**	
Age, y	64 (26-71)^a^
Sex (M/F)	11/5
Time from RTX to HCV therapy, mo	150.9 (84.4)^b^

**Cause of end-stage renal disease**	

Vesicoureteral reflux	1
Chronic glomerulonephritis	6
Others or unknown	9

**HCV genotype**:	

1a	1
1b	10
2	4
4	1

**Baseline laboratory values**	

Creatinine, mg/dL	1.29 (0.80-1.80) ^a^
eGFR, mL/min/1.73 m^2^	60.3 (19.3) ^b^
Proteinuria (g/24 hours)	0.582 (0,768) ^b^
Bilirubin: total, mg/dL	0.58 (0.50-0.80) ^a^
GGT, U/L	47.5 (16-306) ^a^
ALT, U/L	30.2 (11 -137) ^a^
AST, U/L	31.4 (17 -85) ^a^
ALP, U/L	95.1 (41.3) ^b^
Total serum protein, g/dL	6.91 (0.50) ^b^
Albumin, g/dL	4.15 (0.37) ^b^
AFP, IU/mL	2.93 (2.62) ^b^
Hemoglobin, g/dL	12.87 (1.13) ^b^
WBC count, /*μ*L	7.40 (1.57) ^b^
Platelet count, /*μ*L	218.43 (62.16) ^b^
FibroScan value (kPa)	9.31 (1.75) ^b^
Viral load (log_10_ IU/mL)	2.1E^6^ (4.1E^4^-1.3E^7^) ^a^

Data are expressed as median (range)^a^ or mean (standard deviation)^b^. Abbreviations. eGFR: estimated glomerular filtration rate; GGT: *γ*-glutamyl-transpeptidase; ALT: alanine aminotransferase; AST: aspartate aminotransferase; ALP: alkaline phosphatase; AFP: alpha fetoprotein (0-4 IU/ml).

**Table 2:** Scores of SF-36 before starting treatment (BASAL), at the end of therapy (EOT), and 1 year after EOT (n=15).

	**BASAL**	**EOT**	**1 YEAR**	*p value*
Health Change ^a^	50 (25-100)	50 (25-100)	75 (25-100) ^†^	*0.01129*

General Health	42 (28.59)	50.3 (26.69)	47.3 (23.82)	*0.1018*

Physical Functioning ^a^	70 (20-100)	75 (0-100)	90 (0-100)	*0.3359*

Role limitation – Physical ^a^	50 (0-100)	100 (25-100)	100 (0-100)	*0.006152*

Role limitation – Emotional ^a^	33.3 (0-100)	100 (0-100) ^†^	100 (0-100) ^†^	*0.0145*

Social role functioning ^a^	62.50 (25-100)	75 (25-100)	87.50 (25-100) ^†^	*0.08548*

Bodily Pain ^a^	77.5 (22.5-100)	90 (22.5-100)	90 (0-100)	*0.09072*

Emotional well-being ^b^	57.6 (18.57)	70.13 (12.99) ^§^	73.87 (12.82) ^§^	*8e* ^*-04*^

Vitality ^b^	56.3 (22.16)	68 (10.82)	66.67 (20.41)	*0.0807*

Data are expressed as ^a^ median values (range) or ^b^ mean (standard deviation). ^†^ p<0.05 vs BASAL (Friedman's test followed by Bonferroni's correction); ^§^ p<0.05 vs BASAL (ANOVA followed by Tukey post hoc test).

## Data Availability

The data used to support the findings of this study are available from the corresponding author upon request.

## Abbreviations

CLDQ: Chronic Liver Disease questionnaire
DAA: Direct antiviral agents
eGFR: Estimated glomerular filtration rate
EOT: End of treatment
ESA: Erythropoiesis stimulating agents
QOL: Quality of life
SVR: Sustained virological response
TE: Transient elastography.
